# Do We Deliver the Pressures We Intend to When Using a T-Piece Resuscitator?

**DOI:** 10.1371/journal.pone.0064706

**Published:** 2013-05-22

**Authors:** Evelien Roegholt, Jeroen J. van Vonderen, Frans J. Walther, Charles C. Roehr, Arjan B. te Pas

**Affiliations:** 1 Division of Neonatology, Department of Pediatrics, Leiden University Medical Center, Leiden, The Netherlands; 2 Department of Neonatology, Charité University Medical Center, Berlin, Germany; The Ohio State University, United States of America

## Abstract

**Background:**

A T-piece resuscitator (TPR) uses a built-in manometer to set the inflation pressures, but we are not informed what pressures are actually delivered distally. Aim of this study was to measure the proximal and distal pressures under different gas conditions when using a TPR.

**Methodology/Findings:**

A test lung was ventilated using a TPR (PIP 25 cmH_2_O, PEEP 5 cmH_2_O) with a gas flow rate of 8 L/min. A) Pressure delivered by six different TPRs was tested. To test variability 20 participants were asked to set PEEP and PIP pressures to 25/5 cmH_2_O. B) PIP and PEEP were measured proximal and distal of the TPR when using standard tubing or heated tubing with or without a humidifier. In experiment A mean (SD) proximal PIP and PEEP of the TPRs were respectively 20.3 (0.3) cmH_2_O (19.9–20.6 cmH_2_O) and 4.9 (0.1) cmH_2_O. When 20 participants set pressures; PIP 26.7 (0.5) cm H_2_O and PEEP 5.9 (0.44) cmH_2_O were measured. Experiment B showed that the decrease of PIP between proximal and distal pressures was not clinically significant. However there was a significant decrease of PEEP using the standard tubing (5.1 (0.1) cmH_2_O proximally versus 4.8 (0.2) cmH_2_O distally; p<0.001) compared to, when using a humidifier with associated tubing and the humidifier turned on, 5.1 (0.1) proximally versus 3.9 (0.2) cmH_2_O distally; (p<0.001).

**Conclusion/Significance:**

The accuracy of the built-in manometer of a TPR is acceptable. Most pressures set proximally are comparable to the actual pressures delivered distally. However, when using tubing associated with the humidifier PEEP decreases distally by 1.1–1.2 cmH_2_O and users should anticipate on this.

## Introduction

For effective neonatal resuscitation at birth appropriate mask technique is necessary for adequate pressure delivery. International resuscitation guidelines recommend preset peak inspiratory pressures (PIP) and positive end-expiration pressures (PEEP) [1]. Although PIP is a poor proxy for the tidal volume provided during positive pressure ventilation (PPV) [2] it is important for creating a functional residual capacity (FRC) at birth. Also there is increasing evidence that PEEP is important for lung liquid clearance and maintaining FRC [3]. These pressures are often administered using a T-piece resuscitator (TPR) device [4], [5]. When using a Neopuff TPR device to deliver the set PEEP and PIP, flexible tubing is attached between the device and face mask or endotracheal tube. However the ribbed inner-surface and length of the tubing can cause some resistance to the gas flow, leading to a decrease in the delivered pressures. The set pressure displayed is measured proximal (at the Neopuff). However we do not know what pressure is actually delivered distally at the patient interface. In our delivery rooms we have recently introduced heated humidified gases for ventilation of newly born preterm infants using a humidifier and longer ventilation tubing with a heater wire In addition, heating and humidifying air changes the gas condition and will influence the dynamic pressures [6]. This could potentially increase the resistance even more. Therefore we hypothesized that the pressure measured distally at the T-piece is lower that the pressures proximally shown on the manometer of the TPR.

Hence, the aim of this study was to measure the proximal and distal pressures under different gas conditions i.e. using standard tubing with cold dry air or heated humidified air using a heated humidifier with associated tubing. Furthermore, we were also interested in the accuracy of the built-in manometers and compared them with a distally placed manometer.

## Methods

Due to the observational character of this study the institutional review board (IRB) of our hospital (Commissie Medische Ethiek, Leids Universitair Medisch Centrum) reviewed our proposal and declared that medical ethical review of this study was not required. All staff members consented verbally to participate in this study.

The experimental study was performed at the Neonatal Intensive Care department of the Leiden University Medical Centre, a tertiary level perinatal care centre in Leiden, the Netherlands. PPV was provided using a Neopuff device (Fisher and Paykel, Wellington, New Zealand). PIP was set to 25 cmH_2_O, PEEP to 5 cmH_2_O, a gas flow rate of 8 L/min was used with medical air according to the local guidelines. For each experiment PPV was given for 30 seconds at a rate of 40/min. A 50 mL Dräger test lung (Dräger, Lübeck, Germany) with a compliance of 0.65 mL/cmH_2_O was attached directly to a endotracheal tube size 4.0 (Mallinckrodt Medical, Athlone, Ireland) to ensure that the ventilation circuit was airtight. We used F&P standard T-piece tubing (RD 1300-10; Fisher & Paykel Healthcare, Auckland, New Zealand, 160 cm) and when a heated humidifier was placed in the circuit (MR 850, F&P Healthcare, Auckland, New Zealand) we used the accompanying T-piece tube set with heated wire (900RD110; Fisher & Paykel Healthcare, Auckland, New Zealand, 190 cm).

Pressures were measured proximally at the Neopuff and distally at the T-piece of the disposable circuit. Pressures were registered using a Florian respiratory function monitor (Acutronic Medical Systems AG, Hirzel, Switzerland). Before the experiment the Florian was calibrated by using two known pressures (5 and 20 cmH_2_O). A pressure sensor was placed within the ventilation circuit at different places. All data was digitized and recorded at 200 Hz using Spectra Physiological software (Grove Medical, London, UK).

**Figure 1:**
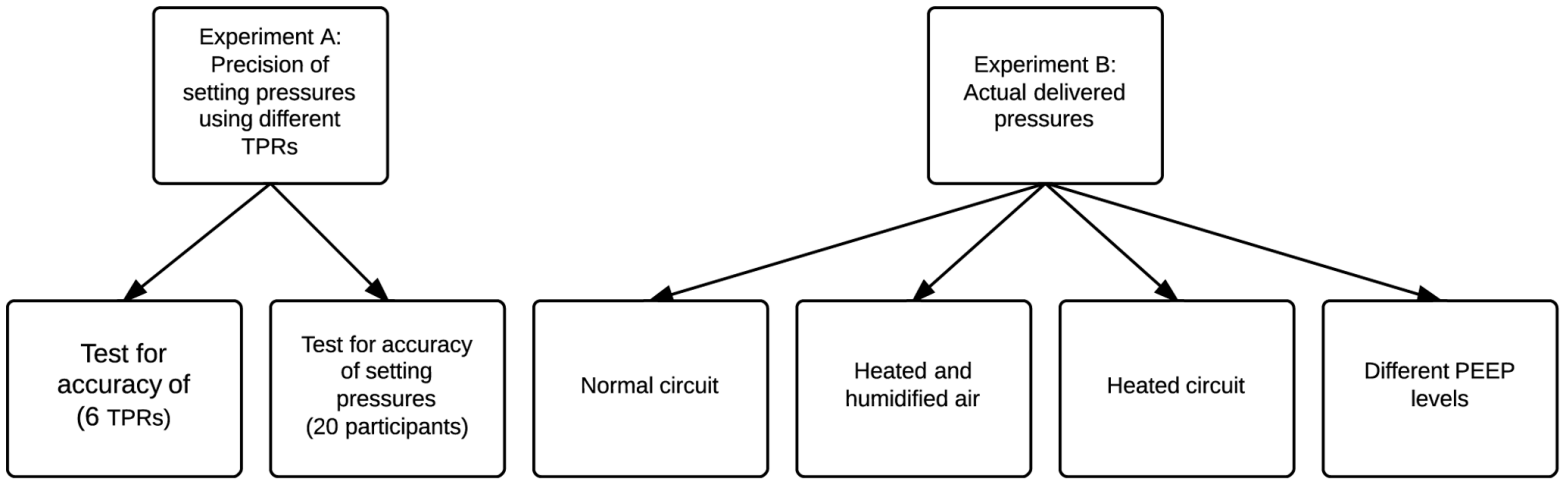
Flowchart of the conducted experiments.

The following measurements were performed (Figure 1):

- To test the accuracy of the manometers of the TPRs available in our unit, we measured pressures proximally at the outlet of all the devices (n = 6) using an industry standard Biotek VT plus gas flow analyser (Biotek Instruments, Winooski, VT, USA). The researcher set the pressures on the TPR device as accurately as possible to PIP 20- and PEEP 5 cmH_2_O. We selected 20 cmH_2_O because this could be set more accurately as there is an indent at 20 cmH_2_O visible on the manometer. After this experiment the manometers of the TPRs were calibrated using the gas flow analyser.

We then tested how accurate pressures could be set by 20 participants on one TPR device. All participants were staff members of our NICU, trained in using the TPR for neonatal resuscitation. Participants were randomly selected from staff on the day of the experiment. Before each measurement it was checked if the dial of manometer on the Neopuff was at zero cmH_2_O when no gas flow was given. The participants were asked to set PIP 25 cmH_2_O and PEEP 5 cmH_2_O as these are the initial pressures recommended in our local guidelines.

**Figure 2:**
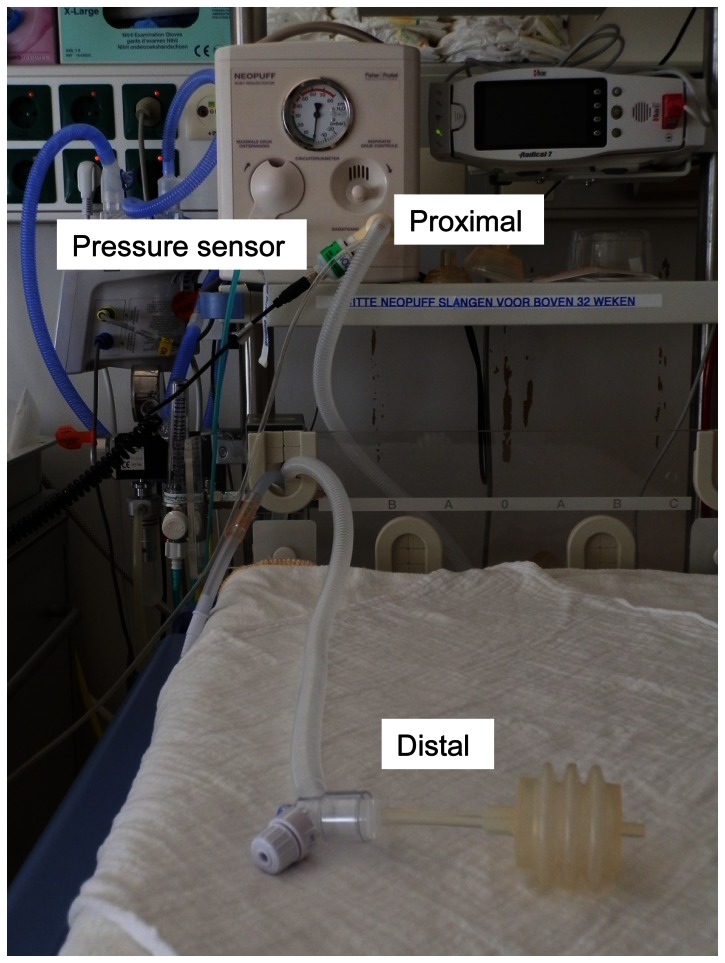
Picture of a T-piece resuscitator device attached to a Neopuff. T-piece resuscitator device with standard tubing attached. Proximal, distal and proximal attachment of the pressure transducer are indicated.

**Figure 3:**
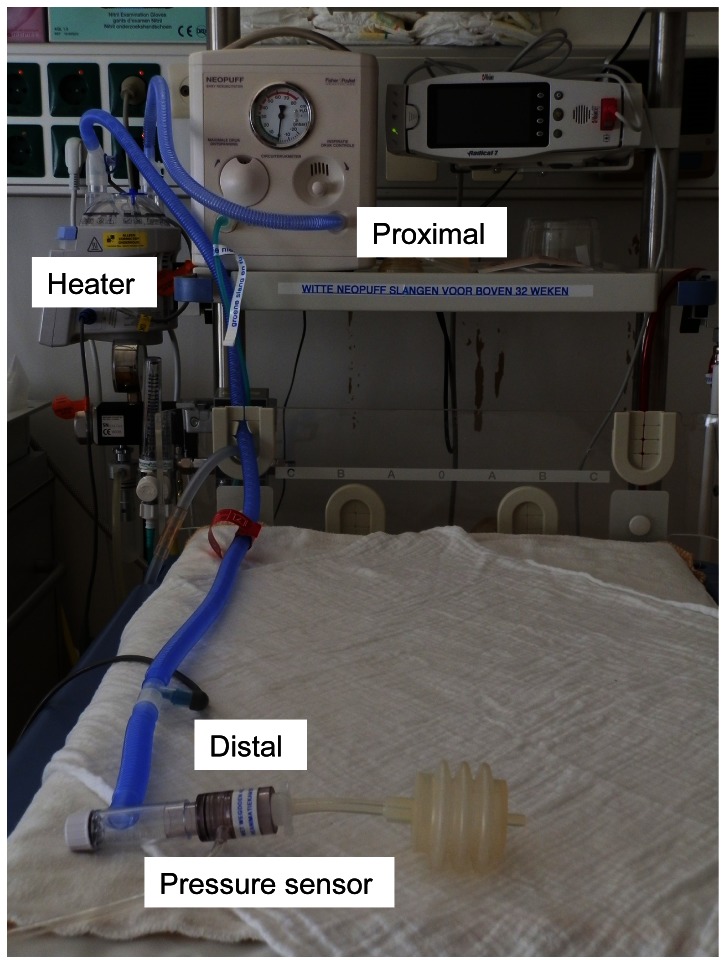
Picture of a T-piece resuscitator device with humidified T-piece circuit attached to a Neopuff. T-piece resuscitator device with humidified T-piece circuit and heater in place. Proximal, distal and distal attachment of the pressure transducer are indicated.

- To evaluate if there was a decrease in pressures delivered we used the calibrated TPR device. Furthermore we evaluated what part of the circuit caused the pressure decrease. The experiment was performed by a neonatologist with more than 10 years of experience in neonatal resuscitation using a TPR. We measured proximal and distal pressures in 4 different set ups:

1. Using standard tubing: proximal (at the Neopuff) and distal (at the end of the TPR) pressures were measured (Figure 2).
2. To evaluate whether heated and humidified air influenced pressures delivered using tubing with heating wire and a humidifier, we compared pressures measured during ventilation of the test lung with A) cold and dry air and B) heated and humidified air (37°C). During measurements with cold and dry air the humidifier was in situ and filled with water and power off. Pressures were measured proximally and distally for both experiments (Figure 3).
3. To evaluate whether a decrease in pressures was caused by the humidifier or by the associated tubing. A) Pressures were measured proximally and distally without a humidifier in place. To assess the influence of the heating wire, which can produce turbulence causing increase resistance and thereby a pressure drop, B) Pressures were measured proximally and distally (at the T-piece) with only the associated distal tubing containing the heating wire (length 130 cm) connected to the Neopuff.
4. To evaluate the amount of pressure decrease after setting different levels of PEEP while ventilating the test lung with the heater turned on. PEEP levels 3, 5, 8 and 12 cmH_2_O were tested at a gas flow rate of 8 L/min.

All experiments were repeated four times to test the consistency in measurements. PIP and PEEP were analyzed for each individual inflation.

### Analyses

Data were analysed using SPSS (SPSS for windows, version 20.0.0, IBM, Chicago, IL, USA). Results are presented as mean (standard deviation (SD)) or median (inter-quartile range (IQR)) where appropriate. Distal pressures were measured to investigate whether the decrease in pressures was caused by the humidifier or by the associated tubing. Pressures were measured with and without a humidifier in place. The differences in pressures were tested using a Wilcoxon rank sum test for unpaired independent non-normally distributed values. A p-value<0.05 was considered as statistically significant. Reported p-values are two-sided.

## Results

### A. Precision of setting pressures using different TPRs

The manometers of the 6 available TPRs were tested. We measured a mean (SD) PIP of 20.3 (0.3) cmH_2_O, and a PEEP of 4.9 (0.1) cmH_2_O.

In total 20 participants set PIP and PEEP pressures using the calibrated TPR. The group consisted of 4 neonatologists with years of experience mean (range) 10 (9–15) years, 2 neonatal-fellows 7 (6–7) years, 3 residents 4 (2–6) years and 11 nurses 9 (2–26) years. We measured a PIP of 26.7 (0.5) cmH_2_O and a PEEP of 5.9 (0.44) cmH_2_O.

### B. Actual delivered pressures

**Figure 4:**
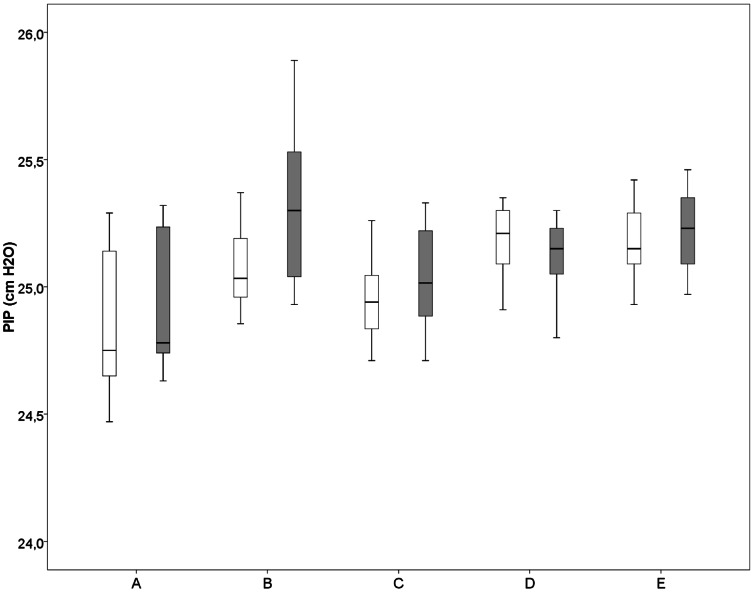
PIP (cmH_2_O) measured at different places in the ventilation circuit. A) standard tubing, B) heater with associated tubing (not heated), C) associated tubing without heating, D) distal associated tubing and E) heater with associated tubing heated. The box plots show median values (solid black bar), inter quartile range (margins of box), and range of data proximal (white boxes) and distal (grey boxes).

**Figure 5:**
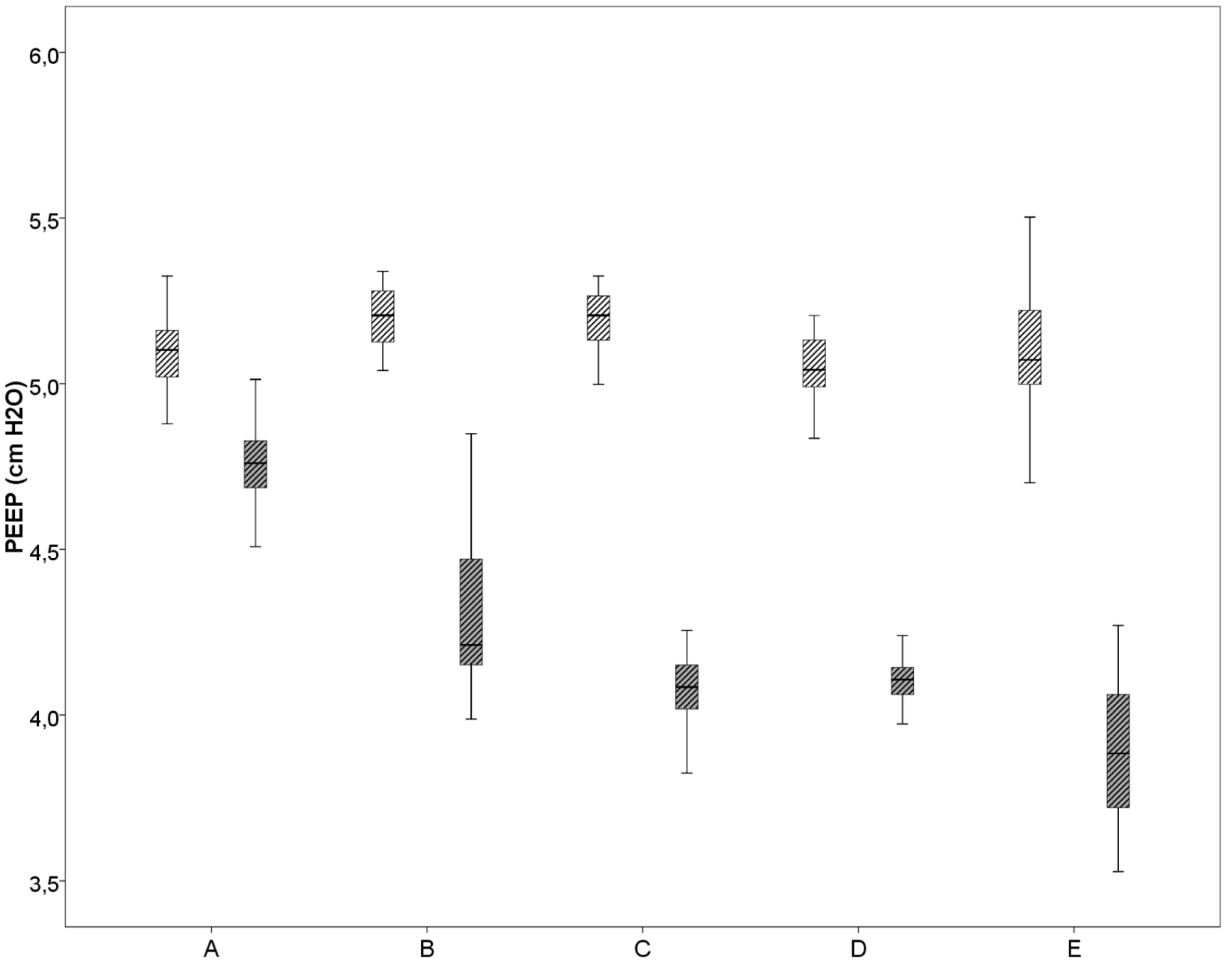
PEEP (cmH_2_O) measured at different places in the ventilation circuit. A) standard tubing, B) heater with associated tubing (not heated), C) associated tubing without heating, D) distal associated tubing and E) heater with associated tubing heated. The box plots show median values (solid black bar), inter quartile range (margins of box), and range of data proximal (white shaded boxes) and distal (grey shaded boxes).

1. When using the standard TPR, The differences in proximal and distal measured pressures (PIP and PEEP) were very small (Table 1, Figure 2).
2. When the humidifier and associated tubing were used and the heater was turned on (i.e. warmed and humidified air) or off (i.e. cold dry air) the difference in proximal and distal measured PIP was very small (Figure 3). This was also the case when the humidifier was not in place or when only distal tubing was measured. However, when comparing proximal vs. distal PEEP, there was 1.0–1.2 cmH_2_O decrease in PEEP distally when humidifier and associated tubing was used, with the heater turned on or off. This decrease remained when the humidifier was not in place or distal tubing was used (Table 1, Figure 4 and 5).
3. Testing 4 different PEEP levels showed a consistent decrease in PEEP measured distally when comparing proximal vs. distal PEEP pressures (Table 2).

**Table 1:** PEEP and PIP measured both distally and proximally for 5 different setups.

Device	Place	Pressure	Median (IQR)	P-value
**Standard tubing**	PIP (cmH_2_O)	proximal	24.8 (24.7–25.1)	<0.05
		distal	24.8 (24.7–25.2)	
	PEEP (cmH_2_O)	proximal	5.1 (5.0–5.2)	<0.001
		distal	4.8 (4.7–4.8)	
**Heated circuit, heater turned of**	PIP (cmH_2_O)	proximal	25.0 (25.0–25.1)	<0.001
		distal	25.3 (25.0–25.6)	
	PEEP (cmH_2_O)	proximal	5.2 (5.1–5.3)	<0.001
		distal	4.2 (4.2–4.7)	
**Heated circuit, without heater**	PIP (cmH_2_O)	proximal	24.9 (24.8–25.1)	<0.05
		distal	25.0 (24.9–25.2)	
	PEEP (cmH_2_O)	proximal	5.2 (5.1–5.3)	<0.001
		distal	4.1 (4.0–4.2)	
**Heated circuit, only distal tubing**	PIP (cmH_2_O)	proximal	25.2 (25.1–25.3)	0.001
		distal	25.1 (25.0–25.2)	
	PEEP (cmH_2_O)	proximal	5.0 (5.0–5.1)	<0.001
		distal	4.1 (4.1–4.1)	
**Heated circuit, heater turned on**	PIP (cmH_2_O)	proximal	25.2 (25.1–25.3)	n.s.
		distal	25.2 (25.1–25.4)	
	PEEP (cmH_2_O)	proximal	5.1 (5.0–5.3)	<0.001
		distal	3.9 (3.7–4.1)	

Pressures measured using a T-piece resuscitator device in 5 different setups at PEEP of 5 cmH_2_O and PIP of 25 cmH_2_O.

**Table 2:** PEEP and PIP measured both distally and proximally at 5 different levels of PEEP.

Device	Place	Pressure	Mean (SD)	P-value
**PEEP 3**	PIP (cmH_2_O)	proximal	24.8 (24.8–24.9)	<0.001
		distal	24.7 (24.6–24.9)	
	PEEP (cmH_2_O)	proximal	3.2 (3.1–3.2)	<0.001
		distal	2.0 (2.0–2.0)	
**PEEP 5**	PIP (cmH_2_O)	proximal	25.2 (25.1–25.3)	n.s.
		distal	25.2 (25.1–25.4)	
	PEEP (cmH_2_O)	proximal	5.1 (5.0–5.3)	<0.001
		distal	3.9 (3.7–4.1)	
**PEEP 8**	PIP (cmH_2_O)	proximal	25.2 (25.2–25.3)	<0.001
		distal	25.3 (25.3–25.3)	
	PEEP (cmH_2_O)	proximal	7.9 (7.8–7.9)	<0.001
		distal	6.8 (6.7–6.8)	
**PEEP 12**	PIP (cmH_2_O)	proximal	25.2 (25.2–25.3)	<0.001
		distal	25.3 (25.3–25.4)	
	PEEP (cmH_2_O)	proximal	11.8 (11.8–11.9)	<0.001
		distal	10.7 (10.7–10.8)	

Pressures measured using a T-piece resuscitator device at PEEP levels of 3, 5, 8 and 12 cmH_2_O and PIP of 25 cmH_2_O. Supplied air was heated and humidified.

## Discussion

The TPR is a common device used for neonatal resuscitation. It is considered easy to use, even for inexperienced operators [7]. When compared to the self inflating and flow inflating bags it is easier to deliver a sustained inflation and continuous positive airway pressure (CPAP) with TPR and delivers the most consistent pressures [7], [8]. The pressures are set according to a built-in manometer, but this will display the proximal generated pressures, but not the actual pressures delivered distally at the patient interface.

We observed only a small variation in proximal pressures delivered by the different TPRs available in our unit. Kelm et al. observed also variability in PEEP levels per TPR, although they reported that all PEEP levels were above the target level while we also observed PEEP levels below the target level [9]. When using an analogue scale less precision and more inter-individual variation in setting the pressures can be expected as it is difficult to set pressures exactly. Perhaps a digital scale incorporated in the device would improve the precision with which pressures can be set considerably.

It is still unknown which level of PIP and PEEP is best to use during neonatal resuscitation [10], [11]. Therefore, it is difficult to know how much deviation for the set PEEP is acceptable and clinically relevant. According to Perlman et al. a PIP of 20–25 cmH_2_O is preferred in preterm infants [5]. The significant differences we observed between set and delivered PIP are not likely to be clinically relevant. However, delivering a PEEP of 3.9 cmH_2_O when 5 cmH_2_O has been set using heated and humidified air might have clinical consequences. Similar pressure differences were found at other PEEP levels. Although we appreciate that the ideal pressures to be delivered are unknown and pressure is a poor proxy for delivered tidal volumes, either way it is important that we actual deliver the pressures we intend to.

The significant decrease in PEEP (1.1–1.2 cmH_2_O) after setting different levels of PEEP, is most likely caused by the heating wire inside the tubing, as we observe similar drops in pressure when the humidifier is not placed in the circuit and also when only the distal tubing is connected to the Neopuff device. The heated wire inside the tube probably causes turbulence and thus increases resistance. Heating air distends and could have compensated for the decrease in PEEP, but this was apparently not sufficient since the pressure drop increases even more when heated and humidified air is used.

When respiratory support at birth is needed PEEP is crucial for lung liquid clearance and maintaining functional residual capacity at birth, especially in preterm infants [3]. Therefore the observed consistent decrease in PEEP by 1.1–1.2 cmH_2_O when humidifier associated tubing is used and heating is turned on could be clinically relevant. Until this has been resorted, the caregiver should anticipate a lower PEEP level when the heated and humidified system is used and set the PEEP level 1 cmH_2_O higher on the manometer.

The differences in decrease between PIP and PEEP can be explained by how the system is pressurized (static or dynamic). When applying a PIP the PEEP- valve at the end of the T-piece is closed and all air flows into the lung until PIP is reached (pressure proximal is equal to pressure distal) and air flow is zero (static pressure). At the end inflation the finger is released, and the PEEP valve is open. PEEP is generated depending on how much air flows through the PEEP valve, generating a dynamic pressure. During PEEP air continues to flow through the tubing, thus more subjective to the resistance of the tube and PEEP is diminished. Also, Hawkes et al described that a change in PEEP can occur after frequent occluding the PEEP valve with the finger could give an unintended turn on the PEEP dial [12]. However, in this way both proximal and distal pressure would change and we did not observe this. Furthermore Fisher and Paykel have improved their T-piece circuit which makes it harder to unintendedly change the PEEP during resuscitation.

All the experiments were performed in vitro, with a test lung and had as sole purpose to test the accuracy of the devices we used for ventilating infants needing support at birth. We are aware that during neonatal resuscitation other issues, such as mask leak and obstruction, will influence the pressures delivered [13]. However, for adequate and effective neonatal resuscitation not only technique but also an accurate device is important.

## Conclusions

We have demonstrated that there are small acceptable differences in pressures delivered between different TPR devices. Larger differences in PEEP arise when the tubing is used, which is associated with the heater. This decrease in PEEP is 1.1–1.2 cmH_2_O and caregivers should anticipate this, because this might influence the effect of respiratory support in preterm infants at birth.
